# Synthesis and Characterization of Manganese-Modified Black TiO_2_ Nanoparticles and Their Performance Evaluation for the Photodegradation of Phenolic Compounds from Wastewater

**DOI:** 10.3390/ma14237422

**Published:** 2021-12-03

**Authors:** Muhammad Irfan, Rab Nawaz, Javed Akbar Khan, Habib Ullah, Tahir Haneef, Stanislaw Legutko, Saifur Rahman, Jerzy Józwik, Mabkhoot A. Alsaiari, Mohammad Kamal Asif Khan, Salim Nasar Faraj Mursal, Fahad Salem AlKahtani, Omar Alshorman, Abdulnour Ali Jazem Ghanim

**Affiliations:** 1 Electrical Engineering Department, College of Engineering, Najran University Saudi Arabia, Najran 61441, Saudi Arabia; srrahman@nu.edu.sa (S.R.); snmursal@nu.edu.sa (S.N.F.M.); fsalkahtani@nu.edu.sa (F.S.A.); omar2007_ahu@yahoo.com (O.A.); 2 Fundamental and Applied Sciences (FASD), Universiti Teknologi PETRONAS (UTP), Bandar Seri Iskandar 32610, Perak, Malaysia; habib_g02832@utp.edu.my; 3 Centre of Innovative Nanostructures and Nanodevices (COINN), Institute of Autonomous Systems, Universiti Teknologi PETRONAS, Bandar Seri Iskandar 32610, Perak, Malaysia; 4 Mechanical Engineering Department, Universiti Teknologi PETRONAS, Bandar Seri Iskandar 32610, Perak, Malaysia; javed.86kn@gmail.com; 5 Department of Civil and Environmental Engineering, Universiti Teknologi PETRONAS (UTP), Bandar Seri Iskandar 32610, Perak, Malaysia; tahirhanifuaf@gmail.com; 6 Institute of Mechanical Technology, Faculty of Mechanical Engineering, Poznan University of Technology, 60-965 Poznan, Poland; stanislaw.legutko@put.poznan.pl; 7 Department of Production Engineering, Faculty of Mechanical Engineering, Lublin University of Technology, 36 Nadbystrzycka Street, 20-618 Lublin, Poland; j.jozwik@pollub.pl; 8 Empty Quarter Research Unit, Chemistry Department, College of Science and Art at Sharurah, Najran University Saudi Arabia, Najran 61441, Saudi Arabia; mabkhoot.alsaiari@gmail.com; 9 Mechanical Engineering Department, College of Engineering, Najran University Saudi Arabia, Najran 61441, Saudi Arabia; mkkhan@nu.edu.sa; 10 Civil Engineering Department, College of Engineering, Najran University Saudi Arabia, Najran 61441, Saudi Arabia; aaghanim@nu.edu.sa

**Keywords:** black TiO_2_ nanoparticles, manganese doping, visible light absorption, bandgap, phenolic compounds, photodegradation, wastewater treatment

## Abstract

The release of phenolic-contaminated treated palm oil mill effluent (TPOME) poses a severe threat to human and environmental health. In this work, manganese-modified black TiO_2_ (Mn-B-TiO_2_) was produced for the photodegradation of high concentrations of total phenolic compounds from TPOME. A modified glycerol-assisted technique was used to synthesize visible-light-sensitive black TiO_2_ nanoparticles (NPs), which were then calcined at 300 °C for 60 min for conversion to anatase crystalline phase. The black TiO_2_ was further modified with manganese by utilizing a wet impregnation technique. Visible light absorption, charge carrier separation, and electron–hole pair recombination suppression were all improved when the band structure of TiO_2_ was tuned by producing Ti^3+^ defect states. As a result of the enhanced optical and electrical characteristics of black TiO_2_ NPs, phenolic compounds were removed from TPOME at a rate of 48.17%, which is 2.6 times higher than P25 (18%). When Mn was added to black TiO_2_ NPs, the Ti ion in the TiO_2_ lattice was replaced by Mn, causing a large redshift of the optical absorption edges and enhanced photodegradation of phenolic compounds from TPOME. The photodegradation efficiency of phenolic compounds by Mn-B-TiO_2_ improved to 60.12% from 48.17% at 0.3 wt% Mn doping concentration. The removal efficiency of phenolic compounds from TPOME diminished when Mn doping exceeded the optimum threshold (0.3 wt%). According to the findings, Mn-modified black TiO_2_ NPs are the most effective, as they combine the advantages of both black TiO_2_ and Mn doping.

## 1. Introduction

TPOME has been reported to contain various phenolic compounds including ferulic acid (dihydroxycinnamic acid, C_10_H_10_O_4_), caffeic acid (3,4 dihydroxycinnamic acid, C_9_H_8_O_4_), gallic acid (3,4,5 trihydroxybenzoic acid, C_7_H_6_O_5_), phenol (C_6_H_5_OH), 3-methylcatechol (C_7_H_8_O_2_), catechol (1,2-dihydroxybenzene, C_6_H_6_O_2_), 4-hydroxybenzoic acid, protocatechuic acid (C_9_H_8_O_3_), p-coumaric acid, and p-hydroxybenzoic acid [1,2]. The phenolic compounds are liberated from palm fruits during the wet extraction processes used in palm oil mills [3]. The total phenolic compounds in TPOME were found to range from 33 to 630 mg/L. The organic pollutant content is significantly higher than the Malaysian Department of Environment’s discharge limit, according to TPOME research (DOE). Because these pollutants are present in such high concentrations, current POME treatment technologies cannot achieve the required standard discharge limit. Chemical pollution from TPOME containing organic and inorganic contaminants could jeopardize aquatic biodiversity, and the presence of hazardous organic components in TPOME could harm aquatic ecology [4]. Because of their high toxicity, inertness, teratogenic and mutagenic behavior, endocrine-disrupting capability, and bioaccumulation potential, phenolic compounds are regarded as a significant threat to aquatic life and human health [2,3]. More effective and robust technologies to extract refractory phenolic compounds from TPOME before discharge into the environment are required to prevent water pollution and maintain the sustainability of palm oil production.

Photocatalysis, according to reports, offers a significant potential for TPOME cleanup and has been well-proven in previous investigations [5,6,7,8,9,10,11,12,13,14]. The immobilized TiO_2_-based photocatalytic technique was able to effectively eliminate more than 90% of COD from TPOME in less than 5 h of UV irradiation [5]. Another work using TiO_2_-based photocatalysis to photo-mineralize TPOME found that 78% of COD was removed from TPOME in 20 h [15]. In the realm of photocatalysis for environmental remediation, nanostructured transition metal compounds with high surface-to-volume ratios, such as TiO_2_, are of particular scientific interest. Despite the increased efficiency and other advantages such as prominent photocatalytic activity, nontoxicity, good corrosion resistance, reduced TiO_2_ cost of production, full calcification, quick reaction rates, and standard temperature and pressure of the procedure [15], published research on photocatalytic activity for TPOME remediation is restricted.

Photocatalysis has been used as a post-treatment approach for the photodegradation of phenolic compounds from TPOM by just a few research groups [16,17]. For example, Nawaz et al. [18] found that 180 min of visible light irradiation removed 48.17 percent of phenolic chemicals from TPOME using black TiO_2_. However, when compared to UV, the visible-light remediation efficiency is relatively poor. The development of visible-light active TiO_2_ is enticing. It is expected to improve the price-efficiency and competence of photocatalysis for polishing TPOME, making the process green and sustainable when employing solar or visible-light irradiation. Palm-oil-producing countries are often found in tropical climates with plenty of sunlight. By reusing the polished effluent, the overall polishing cost can be lowered. As a result, solar-mediated TiO_2_-based photocatalysis is a very appealing approach for the removal of phenolic components from TPOME. Efforts must be made to enhance the effects of the frequently used TiO_2_ photocatalyst so that it can operate better under visible light and create unique alternative means of producing TiO_2_ photocatalyst.

Various research groups have brought about different structural and chemical modifications that can turn white TiO_2_ into various colors such as yellow, blue, gray, red, and black using different starting materials and experimental conditions where the color of the TiO_2_ is dependent on the defect concentration [18,19,20]. Certain chemical and structural modifications including the creation of surface oxygen vacancy (SOVs) or Ti^3+^ species [21], and the formation of surface disorder core-shell structures [22], hydroxyl groups and Ti-H bonding, have been made to produce defective TiO_2_. These modifications are believed to significantly alter the electronic structure and reduce the bandgap of TiO_2_, which results in improved visible-light absorption. Among the various approaches, transition metal doping is thought to considerably improve pollutant adsorption on the surface and drastically improve the photocatalytic capabilities of TiO_2_ [23]. Surface charge density, isoelectric point, band energetics, various oxidation states, electrokinetics, and interfacial electron transfer mechanism all contribute to the co-catalyst effect of transition metals [24,25]. Manganese (Mn) doping is an effective method among the transition metal doping strategies for the following four reasons: (1) Mn doping can improve TiO_2_’s capacity to absorb visible light; (2) trapping charged pairs can improve charge (e^−^/h^+^) pair separation for more extended periods; and (3) producing effective midgap or intermediate bands (IBs) can improve TiO_2_’s adsorption capabilities [26,27]. Studies reported that Mn-doped TiO_2_ functioned well under solar light, eliminating 91.7% of the phenolic compound in 240 min [28]. The influence of Mn doping on the characteristics and implementation of black TiO_2_ NPs for the photocatalytic degradation of phenolic compounds from actual TPOME, on the other hand, is rarely investigated.

Manganese-modified black TiO_2_ (Mn-B-TiO_2_) was synthesized in this study with the help of a modified glycerol-assisted method and a wet impregnation process. X-ray diffraction (XRD), X-ray photoelectron spectroscopy (XPS), diffuse reflectance UV-Visible spectroscopy (DRUV-Vis), photoluminescence spectroscopy (PL), field emission scanning electron microscopy (FESEM) coupled with energy-dispersive x-ray (EDX) analysis, and surface area and porosity measurement were used to characterize the synthesized Mn-B-TiO_2_. The influence of different Mn doping concentrations on the photocatalytic characteristics and performance of black TiO_2_ NPs was investigated. The photocatalytic activity of Mn-B-TiO_2_ was investigated for the photoreduction of phenolic compounds in TPOME under visible light irradiation.

## 2. Materials and Methods

### 2.1. Synthesis of Black TiO_2_ NPs

**Figure 1:**
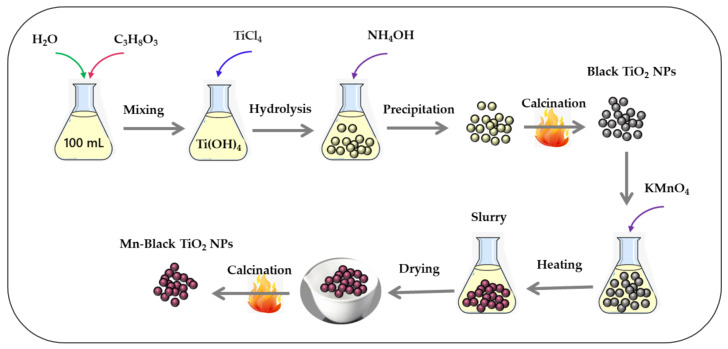
Scheme for the synthesis of black TiO_2_ and Mn-modified black TiO_2_ NPs.

The black TiO_2_ NPs were synthesized via a modified precipitation method by hydrolyzing TiCl_4_, an aqueous glycerol solution, followed by calcination. The precipitation method for the synthesis of defective TiO_2_ was chosen based on its various advantages over other methods: (i) the process can be carried out at ambient temperature; (ii) a homogeneous product is produced; (iii) the calcination temperature is low; (iv) the process is straightforward and offers reasonable control over various properties [29]. The scheme of synthesizing black TiO_2_ photocatalysts is shown in Figure 1. To produce TiCl_4_, an 18.0 mL (0.1 mol) solution was added dropwise to 50 mL of an aqueous glycerol solution with vigorous stirring and an ice-water bath to keep the exothermic reaction temperature under 5 °C. An ammonium hydroxide solution with a concentration of 2.5 M was used in the precipitation procedure, which required the addition of over 300 mL of solution. For the recovery of the TiO_2_ NPs, the pH of the TiO_2_ colloidal suspension was raised to 10, a pH where fast settling and maximum precipitation of the nanoparticles was visually observed. The pH was adjusted to suppress peptization by mild aggregation of the nanoparticles. Once these steps were completed, the samples were washed with distilled water (pH = 7) and centrifuged for 10 min at ambient temperature. Amorphous white TiO_2_ powder was produced by drying the TiO_2_ residue in an oven at 80 °C for 24 h. The dried TiO_2_ powder was ground manually in a mortar and activated by calcination at 300 °C for 1 h to produce black TiO_2_ in the anatase phase. The calcination process was maintained at a heating rate of 5 °C/min for 60 min in a muffle furnace (Nabertherm). The TiO_2_ nanoparticles prepared in this way were labeled as black-TiO_2_.

### 2.2. Synthesis of Mn-Modified Black TiO_2_ NPs

The best performing black TiO_2_ NPs were further modified with manganese (Mn). Mn was chosen as a dopant because Mn, due to having different oxidation states (Mn(II)-Mn(VII)), can act as co-catalyst and plays a crucial role in deciphering the structural, optical, and morphological properties of TiO_2,_ which subsequently influence the photocatalytic performance towards the degradation of various organic contaminants [23]. The Mn-modified black TiO_2_ (Mn-B-TiO_2_) NPs were synthesized via the wet impregnation method. The schematic illustration of the synthesis of defective Mn-B-TiO_2_ is depicted in Figure 1. The stock solution was prepared by dissolving a specific amount of KMnO_4_ in 100 mL of deionized water. Then, specific volumes, such as 1.0 mL, 3.0 mL, and 5.0 mL, of the stock solution were transferred to the TiO_2_ NPs suspension to produce different Mn wt% values such as 0.1, 0.3, and 0.5%, respectively. The solution was heated at 60 °C and vigorously stirred until the slurry was formed. The slurry was concentrated to dryness. The sample was dried at 80 °C for 24 h. The dried sample was calcined at 300 °C for an hour. All the samples were ground and kept in amber glass vials for characterization and further use. The samples prepared in this way were labeled as Mn-B-TiO_2_ (*x*), where *x* is the nominal wt% of Mn, such as 0.1 wt% and 0.5 wt%.

### 2.3. Materials Characterization

Powder X-ray diffraction spectrometer (Rigaku D/max-RA, Rigaku, Austin, TX, USA) patterns were recorded to determine XRD patterns for structural characteristics. This was achieved by employing CuKα radiation (1.5406) at 40 kV and 40 mA in a 20–80° (2) scanning range with a step size of 0.027° to generate the patterns. The average crystallite size and crystallinity of the samples were calculated from XRD data using Equation (1) and Equation (2).

The average crystallite size of the TiO_2_ photocatalysts were calculated according to the Debye–Scherer formula as shown in Equation (1):

(1) $$D=\frac{K\lambda }{\beta \cos\theta }$$

where D is the average crystallite size (nm), K is the Scherer constant which accounts for the shape factor of the particle and is generally taken as 0.9, λ (0.15406 nm) is the wavelength of the incident rays, β is the full width at half maximum (FWHM) of the reflection peak in radian that has the maximum intensity in the diffraction pattern, and θ is the diffraction angle.

The crystallinity of the synthesized TiO_2_ photocatalysts was estimated using the following Equation (2):

(2) $$\mathrm{Crystallinity}\;\left(\%\right)=\frac{\mathrm{Area}\;\mathrm{of}\;\mathrm{crystalline}\;\mathrm{peaks}}{\mathrm{Area}\;\mathrm{of}\;\mathrm{all}\;\mathrm{peaks}}\times 100\%$$

A Transmission Electron Microscope (Tecnai G2-F20 X-Twin TMP, FEI, Hillsboro, OR, USA), with an accelerating voltage of 100 kV, was used for high-resolution transmission electron microscopy (HRTEM). The ESCALAB 250Xi X-ray photoelectron spectrometer (Thermo Fisher Scientific, Waltham, MA, USA) was used to conduct XPS studies. Lasers were utilized to excite the sample. The instrument was calibrated using a C1s correction of 284.6 eV. Diffuse reflectance UV-Visible spectra were acquired using BaSO_4_ as a standard material with a UV-Vis spectrometer (Agilent Carry100, Santa Clara, CA, USA).

The bandgap energies of the synthesized TiO_2_ photocatalysts were determined from reflectance (F(R)) spectra using the KM (Kubelka–Munk) function [30], as given in Equation (3):

(3) $${\left[F\left(R\right)\mathrm{hv}\right]}^{1/2}=\;K\left(\mathrm{hv}-E_{g}\right)$$

where K is the constant and a characteristic of the semiconductor, hv represents the corresponding photon energy, and Eg is optical bandgap energy. The indirect bandgap was obtained by Tauc’s plot method [31] where [F(R)hv]^1/2^ was plotted versus the hv and the linear portion of the graph was extrapolated to the energy (hv) axis.

PerkinElmer LS–55 fluorescence spectrophotometer (Inc., Waltham, MA, USA) was used to record the PL spectra of the samples. An ASAP 2020 analyzer was used to measure the physio-sorption isotherms of the materials at −196 °C. When analyzing the adsorption data, the Brunauer–Emmett–Teller (BET) techniques were used to calculate the sample-specific surface area (SSA). The pore size was estimated using the Barrett–Joyner–Halenda (BJH) method.

### 2.4. Photocatalytic Activity Evaluation

Laboratory scale photocatalytic reactions were performed in batch mode. A 250-mL Pyrex^®^ photoreactor (Merck, Darmstadt, Germany) (internal diameter, 7.0 cm; height, 9.0 cm), with a quartz window that was firmly sealed at the top to prevent evaporation of the working TPOME solution, served as the experimental apparatus. Throughout the tests, the TPOME’s working capacity was kept constant at 50 mL. As the visible light source, a 500 W halogen bulb was placed 10 cm above the photoreactor. The halogen lamp produced a continuous spectrum of light from near UV to the infra-red region. However, most radiation was from the visible range (λ = 400–750 nm). Photocatalytic reactions were performed to evaluate the performance of black TiO_2_ and Mn-B-TiO_2_ NPs in terms of the degradation of phenolic compounds from TPOME under visible-light irradiation. An accurately weighed amount of each TiO_2_ sample was suspended in 50 mL of TPOME solution to produce 0.8 g/L of its concentration. All the experiments were conducted at a fixed loading of the photocatalyst. Other factors were held constant to avoid the influence of extraneous factors and to obtain specific results of the photocatalytic performance of the synthesized TiO_2_ NPs. To achieve adsorption–desorption equilibrium, the suspension was agitated for 30 min in the dark. Afterward, the rest was exposed to visible light for a total of 180 min. A 1.0 mL aliquot was taken out of the photoreactor for 180 min using a high-precision syringe. A Captiva Econo Filter (Nylon 0.2 m, Santa Clara, CA, USA) filtered each sample before analysis.

Before and after the treatment, the spectrophotometric concentration of phenolic compounds was measured using a modified Folin–Ciocalteu (F–C) analytical method. F–C phenol solution at a quantity of 0.5 mL was diluted with 0.1 mL of a five-fold diluted TPOME sample. The sample was maintained at room temperature for an hour after adding 0.5 mL of Na_2_CO_3_ (200 g/L). This was followed by 765 nm absorbance measurements using a SpectroVis Plus spectrophotometer (Vernier, Beaverton, OR, USA). The absorbance measurements were then transformed into phenolic compounds using the gallic acid standard calibration curve and expressed in mg/L of GAE. To obtain the standard calibration curve, gallic acid solutions of various concentrations (10 to 50 mg/L) were prepared by serial dilution. The corresponding absorbance reading was carried out within the limits of the calibration curve. The concentration of phenolic compounds was taken into consideration as total phenolic compounds (TPC) and expressed as mg/L of gallic acid equivalent (GAE). The F–C reagent contained phosphomolybdic and phosphotungstic complexes. This relied on the transfer of electron in alkaline medium from phenolic compounds to form a blue chromophore constituted by phosphomolybdic and phosphotungstic complexes, where the maximum absorbance depended on the concentration of phenolic compounds. The removal efficiency of the phenolic compounds (GAE) was calculated using Equation (4):

(4) $$X\;\left(\%\right)=\frac{C_{i}-C_{t}}{C_{i}}\times 100\%$$

where X (%) is the degradation efficiency of the phenols after a specific interval of 30, 60, 120, and 180 min of reaction, C_i_ is the initial concentration, and C_t_ is the concentration of phenolic compounds at a specific time interval.

### 2.5. Kinetics and Electrical Energy Consumption

Understanding the mechanism and performance of a system is aided by determining the rate of reactions in reaction studies [26]. A pseudo-first-order kinetic model expression, Equation (5) [27], was used to evaluate the TPOME remediation effectiveness in the removal of phenol l from TPOME by various TiO_2_ NPs.

(5) $$\log\left(\frac{C_{i}}{C_{t}}\right)=-k_{\mathrm{app}}t$$

where C_i_ and C_t_ are the initial concentration before treatment and the concentration of phenols (ppm) at any given time during the treatment, respectively, k_app_ (min^−1^) is the apparent rate constant, and t is the reaction time (min).

The electrical energy consumed (EE) in kWh/m^3^ to reduce the phenol concentration by one order of magnitude per unit volume of phenolic wastewater under visible light irradiation was estimated according to the following Equation (6) [32]:

(6) $$\mathrm{EE}=\frac{1000\times P_{t}}{V\times \log\left(\frac{C_{o}}{C_{t}}\right)}$$

P_t_ is the applied electrical power (kW); t is the treatment time (h) required to treat a specific volume (V) of wastewater (L); 1000 is the conversion factor used to convert grams to kilograms; and C_i_ and C_t_ are the initial and concentrations of phenols at any sampling interval, respectively. EE is the required electrical energy to treat a unit volume (V) of phenol solution.

## 3. Results and Discussion

### 3.1. Properties of the Synthesized Materials

**Figure 2:**
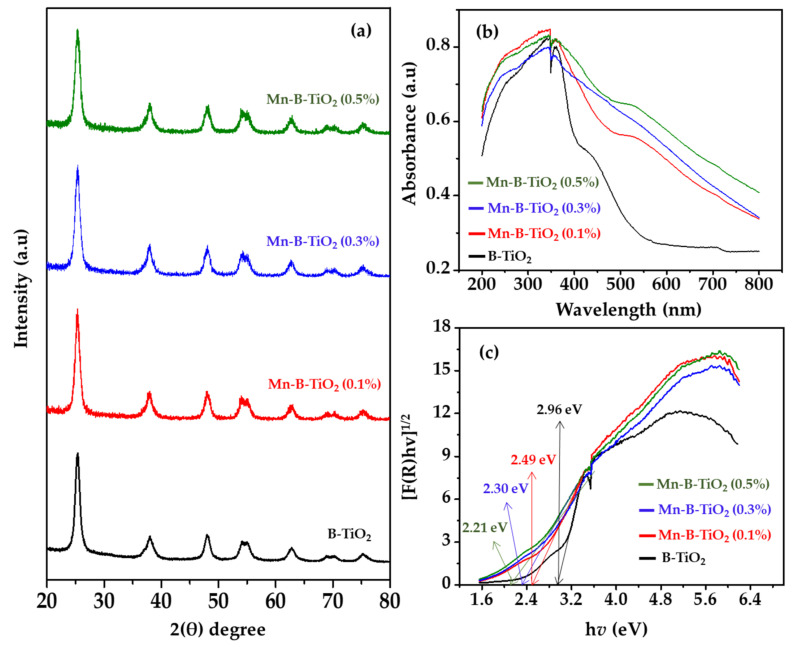
(**a**) XRD patterns, (**b**) DRUV-Vis spectra, and (**c**) Tauc’s plot of the bandgap energies of black and Mn-modified black TiO_2_ NPs.

The XRD patterns of B-TiO_2_ and Mn-B-TiO_2_ NPs, with various concentrations of Mn, are presented in Figure 2a. The XRD patterns of B-TiO_2_ and Mn-B-TiO_2_ NPs showed characteristic diffraction peaks at 2θ = 25.2°, corresponding to the anatase crystalline phase, which agrees well with the previous investigations [33]. No additional peaks associated with other steps such as rutile and brookite were observed for the Mn-modified samples, indicating that the pure anatase phase is essentially maintained after Mn doping. The XRD analysis revealed that the crystal structure of the samples doped with Mn remained unchanged. Furthermore, the diffraction peaks corresponding to crystalline Mn_2_O_3_ or Mn_3_O_4_ were not detected in the XRD pattern of Mn/TiO_2_, which is consistent with previous work [26]. Manganese oxides were not seen in the bulk within the detection limit of the x-ray diffractometer employed in the present work. This could be due to the low concentration of Mn dopant (circa, 0.5 wt%) and the replacement of the Ti ion with the Mn element in the TiO_2_ matrix due to almost similar ionic radii of Ti^4+^ (0.60 Å) and Mn^4+^ (0.53 Å) or the low calcination temperature, which could not reach the thermodynamic driving force [34]. The average crystallite size, crystallinity, and lattice parameter, such as the d-spacing of the undoped and Mn-doped TiO_2_, are presented in Table 1. The average crystallite size increased and the crystallinity decreased with the increasing of the Mn concentration. The crystallite sizes of Mn-TiO_2_ increased in the current study, consistent with previous work [34]. In contrast, Ma et al. [35] reported a decrease in the crystallite size of TiO_2_ with the increasing of the Mn doping level. The crystallinity decreased from 62.89 to 45.79% upon the increasing of the Mn concentration to 0.5 wt%.

The DRUV-Vis absorption spectra were collected for the B-TiO_2_ and Mn-B-TiO_2_ NPs and are shown in Figure 2b. Mn doping did change the absorption edge, and a distinct enhancement of visible-light absorption was observed in Mn-B-TiO_2_ NPs. The primary absorption edge of the B-TiO_2_ NPs was around 400 nm, representing the UV region, which corresponds to the charge transfer between O^2−^ and Ti^4+^ [36]. In contrast, its secondary absorption edge was well shifted to the visible light region (>500 nm). At first, the absorption edge was redshifted to 450 nm at 0.1 wt% Mn doping of B-TiO_2_ NPs. A massive redshift of the absorption edge was achieved at 0.3 wt% Mn doping. Further increasing the Mn doping level to 0.5 wt% showed no apparent redshift of the absorption edge, though the absorption intensity was intensified. Binas et al. [37] investigated the effect of Mn doping on the optical properties of TiO_2_. In their report, a similar redshift of the visual absorption edge into the visible region at 0.1 wt% Mn was demonstrated. The intensity of the absorption band became more robust with the increasing of the Mn doping concentration, suggesting improved visible light absorption by Mn-B-TiO_2_ NPs.

A charge transfer between Mn (d electrons) and CB or VB (i.e., d–d transitions in the crystal field) may explain the redshift of the absorption edge and the amplification of the visible-light absorption [38]. The Mn doping of TiO_2_ significantly improves visible-light harvesting, according to the data. At 0.5 wt percent, optical absorption was extended into the spectrum’s visible and even infrared ranges. The absorption of visible and infrared light might reduce the bandgap and the formation of several midgap states in defective-Mn/TiO_2_. For TiO_2_, Mn doping creates an extra bandgap-occupied state because of the interaction between the t 2 g state of the Mn and Ti atoms. It is the spin polarization of an unpaired valence electron that creates the intermediate bands [39]. For this reason, the formation of intermediate bands in Mn-doped materials is facilitated by the orbital occupancy. Degenerate 2D states of doped Mn are responsible for the extra curved medium bands [36]. Such bars serve as the stepping-stones to enhance the effective optical absorption of the Mn-B-TiO_2_ NPs.

**Figure 3:**
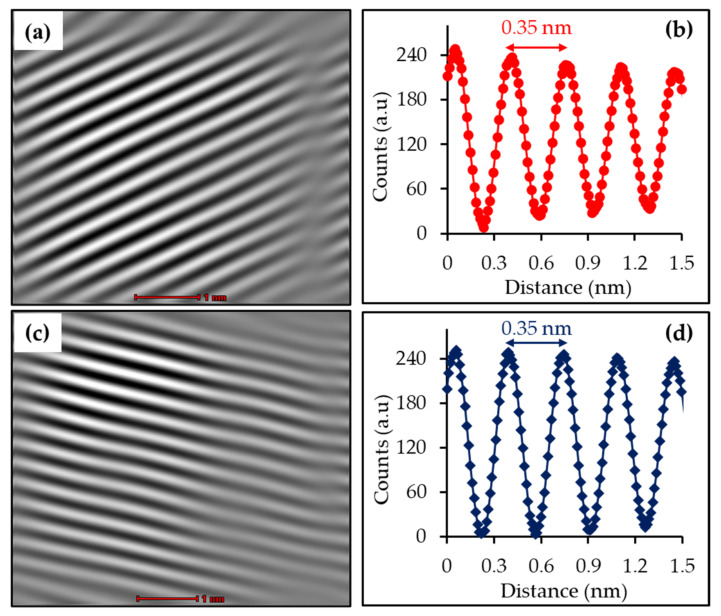
HRTEM images of (**a**) B-TiO_2_ and (**c**) Mn-B-TiO_2_ NPs and line profiles of (**b**) B-TiO_2_ and (**d**) Mn-B-TiO_2_ NPs.

The bandgap energies for B-TiO_2_ and Mn-B-TiO_2_ NPs are presented in Figure 3c. The results suggested the incorporation of Mn into the host lattice, which decreased the bandgap of TiO_2_. The bandgap was dramatically lowered from 2.96 to 2.49 eV at 0.1 percent Mn concentration, while the bandgap was further reduced to 2.30 and 2.21 eV with 0.3 and 0.5 wt percent Mn, respectively. According to previous research, new impurity energy levels or intermediate bands were created in the semiconductor’s bandgap to reduce the bandgap [37]. According to Momeni et al. [40], the decrease in the bandgap of Mn/TiO_2_ may have been due to the augmented electrons present in the form of Mn ions, which resulted in the hybridization of the d-states of Mn with the CB edge of the host. This hybridization led to the extension of VB maximum and the downshifting of the CB minimum with an effective narrowing of the bandgap of TiO_2_. As can be observed, the bandgap of the Mn-B-TiO_2_ NPs monotonically decreased with the increase in the Mn dopant concentration. As reported in the literature, this could have been due to the enhanced interaction of the electron in the d-states of Mn with the host CB electron, which resulted in a massive narrowing of the bandgap [41].

**Figure 4:**
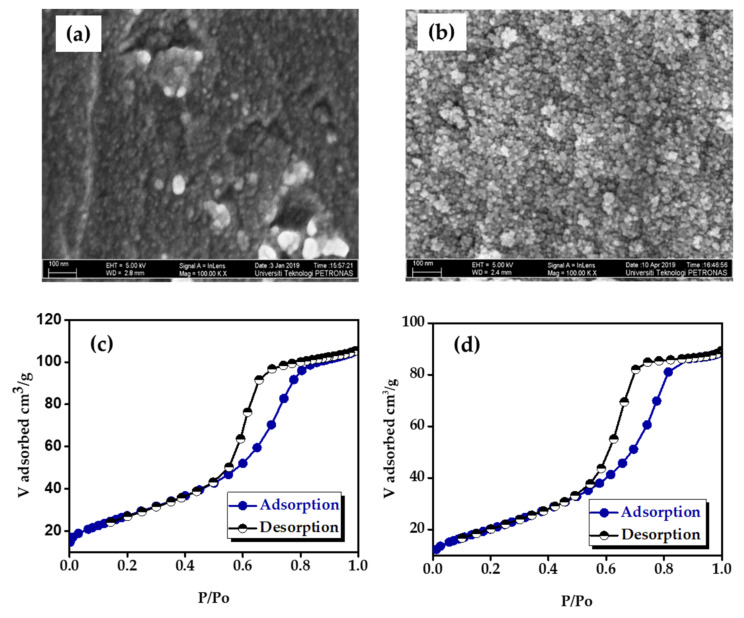
FESEM images of (**a**) B-TiO_2_ and (**b**) Mn-B-TiO_2_ NPs and N_2_ adsorption–desorption isotherms of (**c**) B-TiO_2_ and (**d**) Mn-B-TiO_2_ NPs.

The morphologies of the B-TiO_2_ and Mn-B-TiO_2_ NPs were examined via HRTEM and FESEM. The HRTEM images of B-TiO_2_ and Mn-B-TiO_2_ NPs at the 1 nm scale were obtained, and the results are shown in Figure 3a,b, respectively. It can be seen that both the B-TiO_2_ and Mn-B-TiO_2_ NPs presented well-resolved lattice fringes throughout the surface and the distance between the two adjacent lattice planes was 0.35 nm (Figure 3b,d), which was typical of anatase TiO_2_. The HRTEM results were consistent with XRD results confirming the anatase of the samples. The FESEM images of the B-TiO_2_ and Mn-B-TiO_2_ NPs captured at 100 k× magnification are shown in Figure 4a,b, respectively. All the Mn-doped samples showed spherical shapes with well-defined grain boundaries. It is important to note that the B-TiO_2_ NPs prepared in aqueous glycerol showed a slightly loose agglomeration of the particles (Figure 4a).

Nevertheless, the surface morphology was ameliorated, and the particles were found in a spherical shape with well-defined grain boundaries when doped with Mn (Figure 4b). It is likely that Mn doping had a positive effect on the surface morphology of the TiO_2_ nanoparticles, where the accumulation of the particles was reduced after the Mn doping. No other observable changes, such as the transition from spherical shapes to different shapes, were detected upon Mn doping. The original spherical shape of the particles was retained at an even higher Mn doping level (0.5 wt%).

The N_2_ adsorption–desorption isotherms of the B-TiO_2_ and Mn-B-TiO_2_ NPs, with different Mn concentrations, are shown in Figure 4a,b, respectively. Both the B-TiO_2_ and Mn-B-TiO_2_ NPs showed type-IV isotherms with H_2_-type hysteresis loops. The isotherms indicated that Mn doping did not change the mesoporous nature of the TiO_2_ samples. The only noticeable change observed was that the Mn-B-TiO_2_ NPs show lower N_2_ adsorption than B-TiO_2_ NPs. Compared to B-TiO_2_ NPs, Mn-B-TiO_2_ NPs with various concentrations of Mn showed a less steep desorption branch. The inflection points of Mn Mn-B-TiO_2_ NPs did not show any noticeable shift of the inflection point. The change in the inflection point indicated a higher loading rate. The textural properties, including BET specific surface area, pore-volume, and pore size of the B-TiO_2_ and Mn-B-TiO_2_ NPs, are listed in Table 1. The surface area for both Mn-B-TiO_2_ NPs showed a decreasing trend with the increasing of the Mn dopant concentration. The surface area of B-TiO_2_ NPs was reduced to 97.35, 94.03, and 74.60 m^2^/g at 0.1, 0.3, and 0.5 wt% Mn doping concentration, respectively. Mn-B-TiO_2_ NPs showed a total surface area reduction by 25.32% when the Mn doping concentration was increased to 0.5 wt%. The results indicated that the surface area was reduced with Mn doping due to partial pore blockage by Mn, which was consistent with previous work [23]. In contrast, previous works reported that the surface area increased with the increasing of the Mn concentration [35]. It should be noted that the surface area of all the Mn-doped samples in the current study was higher than the commercial TiO_2_ (50 m^2^/g). Unlike the surface area, the pore volume of Mn-B-TiO_2_ NPs increased from 0.198 to 0.231 cm^3^/g. This indicates that the surface area was reduced with Mn doping due to partial pore blockage by Mn. Irrespective of the Mn doping concentration, Mn-B-TiO_2_ NPs showed a narrower pore size (<8 nm) than P25. The pore size was mainly distributed between 3 and 6 nm. Notably, the obtained pore size distribution indicated that the undoped and Mn-doped samples were homogeneous with uniform pore size distribution ranging from 2 to 8 nm.

**Figure 5:**
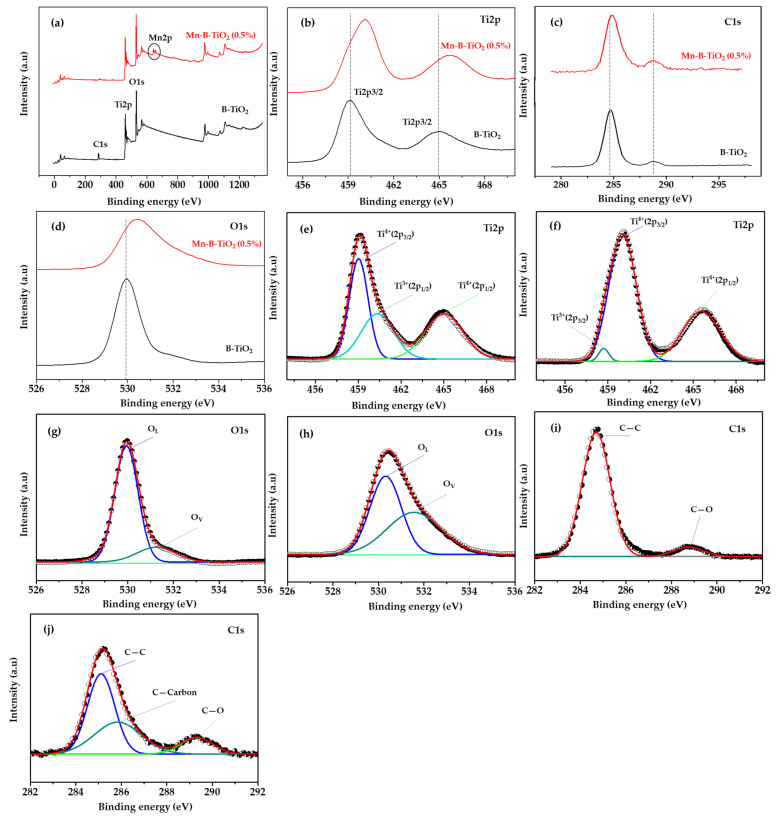
Comparison of (**a**) XPS survey spectra, (**b**) Ti2p spectra, (**c**) C1s spectra, and (**d**) O1s spectra; and deconvoluted Ti2p spectra of (**e**) B-TiO_2_ (**f**) Mn-B-TiO_2_; deconvoluted O1s spectra of (**g**) B-TiO_2_ (**h**) Mn-B-TiO_2_; deconvoluted C1s spectra of (**i**) B-TiO_2_ (**j**) Mn-B-TiO_2_ NPs.

XPS analysis was performed to obtain information on the chemical composition and electronic states of the elements present in B-TiO_2_ and Mn-B-TiO_2_ NPs. Figure 5b shows a comparison of the Ti2p XPS spectra of B-TiO_2_ and Mn-B-TiO_2_ NPs. As shown in Table 2, the orbital doublet peaks Ti2p_3/2_ and Ti2p_1/2_ of the Mn-B-TiO_2_ NPs were both located at a higher binding energy (BE) of 460.04 and 465.58 eV compared to 459.04 and 465.05 eV, respectively. These peak positions were typical of anatase phase TiO_2_ [42]. The results suggested that the anatase phase of the synthesized Mn/TiO_2_ was intact after Mn doping, which is consistent with the XRD results presented in Figure 2. To further analyze the samples, the deconvoluted Ti2p XPS spectra of the B-TiO_2_ and Mn-B-TiO_2_ NPs were compared as shown in Figure 5e,f, respectively. Shoulder peaks at BE of 460.46 and 458.70 eV were observed in the B-TiO_2_ and Mn-B-TiO_2_ NPs, respectively, attributed to Ti^3+^ species. The shift of the Ti2p peaks to higher BE suggested the modification in the coordination environment of Mn-B-TiO_2_ NPs. It is essential to mention that the positive shift of Ti2p_3/2_ peak to high BE indicated lattice distortion due to the incorporation of Mn into the TiO_2_ matrix. It should be noted that the Ti^3+^ defect states were also present in the 0.5% Mn B-TiO_2_ NPs, as indicated by the presence of a broader peak at a higher BE of 460.59, highlighted in green color.

The O1s spectra of B-TiO_2_ and Mn-B-TiO_2_ NPs were compared in Figure 5d. A similar positive shift in O1s spectra was also observed for Mn-B-TiO_2_ NPs. The deconvoluted O1s spectra of the two samples are shown in Figure 5g,h. The B-TiO_2_ and Mn-B-TiO_2_ NPs presented two overlapping peaks at BE 529.94, and 531.37 eV, attributed to lattice oxygen and oxygen deficient region (O_v_), respectively [43]. The deconvoluted O1s spectra proved that the oxygen vacancies were created by Mn doping and agreed well with previous studies [44,45] that reported similar results in terms of oxygen vacancy generation by Mn doping.

The C1s spectra of the B-TiO_2_ and Mn-B-TiO_2_ NPs are compared in Figure 5c. The C1s spectra of both samples showed two peaks where the C1s peaks of the Mn-B-TiO_2_ NPs showed a positive shift to higher BE. The two peaks corresponding to C—C and C—O were located at 284 and 288.78 eV in the B-TiO_2_ NPs, and that of the Mn-B-TiO_2_ NPs shifted to 285.08 289.24 eV, respectively. The deconvoluted C1s spectra of the B-TiO_2_ NPs and the Mn-B-TiO_2_ NPs are shown in Figure 5i,j, respectively. The peaks at lower BE were adventitious elemental carbon and the other was COO. Compared to B-TiO_2_ NPs, the C1s spectra of the Mn-B-TiO_2_ NPs revealed three peaks, and the peak at a BE of 285.88 can be attributed to coke carbon coming from the glycerol in the synthesis medium of the native TiO_2_. The coke carbon contributed to the enhanced visible light response of the Mn-B-TiO_2_ NPs.

**Figure 6:**
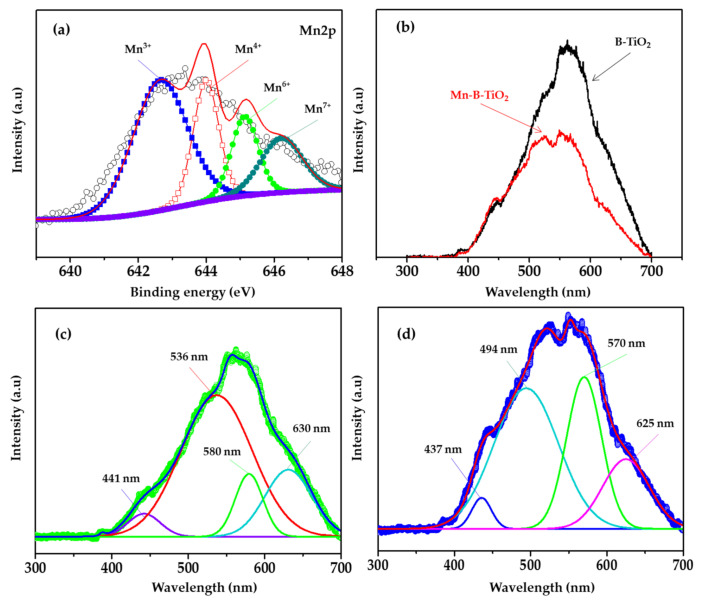
(**a**) Mn2p XPS spectra of Mn-B-TiO_2_ NPs, (**b**) PL spectra, and (**c**) deconvoluted PL spectra of B-TiO_2_ and (**d**) Mn-B-TiO_2_ NPs.

The presence of Mn in the host TiO_2_ matrix was confirmed by the XPS survey spectra presented in Figure 5a. Mn can display six different oxidation states (0, 2+, 3+, 4+, 6+ and 7+), and the Mn 2p_3/2_ electronic states from XPS analysis were documented as follows. The theoretical BE value or peak position of Mn^0^ (metal) was 638.8 eV [46], and Mn^2+^ (MnO) was located at a rather higher BE of 640.97 eV [47]. Mn^3+^ (Mn_2_O_3_) and Mn^4+^ (MnO_2_) appeared at an even larger BE of 642.0 and 643.41 eV [48]. Mn^6+^ (K_2_MnO_4_) and Mn^7+^ (KMnO_4_) peaks were located at 644.2 and 645.6 eV, respectively [46]. Representative deconvoluted high resolution XPS spectra of 0.5 wt% Mn-B-TiO_2_ are displayed in Figure 6a. The Mn2p XPS spectra showed two symmetrical spin-orbit doublet peaks arising from M2p_3/2_ and Mn2p_1/2_. The M2p_3/2_ peak was deconvoluted into four peaks using Gaussian line fitting. The experimental peak positions were 642.65, 643.98, 645.41, and 646.28 eV, which were attributed to the Mn^3+^, Mn^4+^, Mn^6+^, and Mn^7+^ states of manganese due to good agreement with the XPS spectra reported in the literature [26]. The Mn2p_3/2_ peaks were assigned to the surface Mn^3+^ (642.91 eV) due to good agreement with the reported value of 642.4 eV [48,49]. Other peaks at higher BE values of 643.73, and 645.30 eV could thus be attributable to the higher valence state of Mn, e.g., Mn^4+^ [50,51,52]. These result was consistent with the theoretical prediction by Shao et al. [53], which demonstrated that charge transfer happens between substitutional Mn and the host Ti atoms, and results in the lowering of the oxidation state of the Mn atom from M^7+^ to Mn^3+^ and Mn^4+^. No signature peak (640.8 eV) corresponding to Mn^2+^ [52] was observed, suggesting that the Mn is only present in Mn^3+^, Mn^4+^, and higher oxidation states. The coexistence of Mn^3+^ and Mn^4+^ on the surface of Mn-B-TiO_2_ has an important impact on the formation of surface oxygen species, because the mixed-valence state of metal can promote oxygen mobility in the semiconductor oxides [23,28].

Since the photocatalytic performance of TiO_2_ relies on the duration of excited electrons and holes on its surface before they are annihilated by recombination, the PL spectra of the TiO_2_ and Mn-B-TiO_2_ NPs were, therefore, examined to investigate the recombination of electron–hole pairs. An excitation wavelength of 325 nm yielded emission spectra with wavelengths between 300 and 700 nm. The PL spectra of both the B-TiO_2_ and Mn-B-TiO_2_ NPs are shown in Figure 6b. The prominent emission peaks of the B-TiO_2_ NPs emerged at 441, 536, 580, and 630 nm, corresponding to bandgap energies of 2.81, 2.31, 2.13, and 1.96 eV, respectively. In the bandgap transition, the peak at 441 nm corresponded to the anatase phase bandgap energy. The cliffs at 446 were attributed to the free excitation of the band-edges [43]. It should be emphasized that the intensities of the PL peaks of the Mn-B-TiO_2_ NPs were significantly reduced compared to those B-TiO_2_ NPs. The Mn-B-TiO_2_ NPs showed a significant reduction in photoexcited electron and hole recombination, according to the findings. The introduction of Mn may be to blame for the reduced spectral intensity. Generally, the low recombination rate of electron–hole pairs helps to improve photocatalytic performance.

Below is another interesting comparison between the TiO_2_ sample prepared with and without glycerol. A Gaussian line was fitted to the experimental PL results of the B-TiO_2_ NPs and Mn-B-TiO_2_ NPs. The recombination rate induced could be explained based on the emission of the characteristic deconvoluted peaks and their intensities. The intensities of the peaks and their FWHM clearly showed the different quality of both samples. Four prominent emission peaks could be observed at 441, 536, 580, and 630 nm for B-TiO_2_ NPs in Figure 6c, which could be self-trapped excitation, blue emission due to the recombination of free electrons and trapped holes, and the recombination of free spots and trapped electrons (red emissions), respectively [54]. On the other hand, the B-TiO_2_ NPs showed three emission bands at 437, 494, 570, and 625 nm, as depicted in Figure 6d.

According to Wang et al. [55], the PL in black TiO_2_ nanomaterials may a follow donor-acceptor mechanism. After that, SOV/Ti^3+^ captures free electrons, and surface OH groups capture photogenerated holes. The green emission around 536–580 nm could be due to the radiative recombination of the hole trap and the photoexcited electron in the conduction band [56]. The blue-shifted peak around 625−630 can be attributed to the radiative recombination of electrons trap and holes in the valence band [57]. Radiative recombination occurs when the electron in the CB recombines with the hole in the VB, and the excess energy is emitted in the form of a photon. It is the radiative shift of an electron in the CB to an empty hole in the VB [58]. PL analysis suggests that the e^−^/h⁺ pair recombination was considerably halted in the Mn-B-TiO_2_ NPs.

### 3.2. Photocatalytic Performance

**Figure 7:**
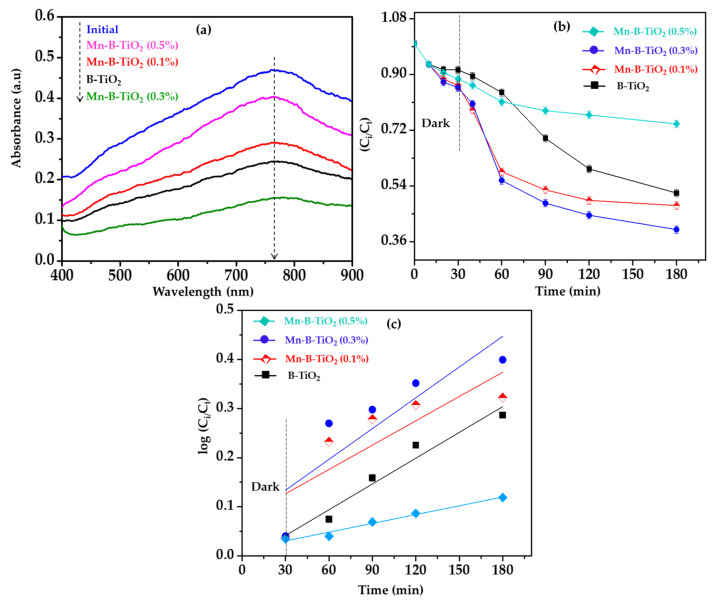
(**a**) UV-Vis absorbance spectra of TPOME after treatment **(b)** photocatalytic performance of B-TiO_2_ and Mn-B-TiO_2_ NPs for phenols’ removal and (**c**) kinetic plots of photocatalytic phenols’ removal.

B-TiO_2_ and Mn-B-TiO_2_ NPs were tested for their ability to degrade TPOME phenolic compounds under visible light irradiation to determine their efficacy. The UV-Vis absorbance spectra of the TPOME solution after the treatment is shown in Figure 7a. The intensity of the maximum absorbance peak shows a clear depreciation, suggesting the removal of phenols by the B-TiO_2_ and Mn-B-TiO_2_ NPS. To quantify the removal of phenols, the absorbance data obtained were compared with a standard calibration curve. The normalized concentration (C/C_o_) of phenolic compounds vs. time after 180 min of visible-light irradiation in the presence of B-TiO_2_ and Mn-B-TiO_2_ NPs is shown in Figure 7b. Controlled experiments performed in the dark and in the presence of NPs showed a removal of less than 12% of phenolic compounds by adsorption. A noticeable improvement in phenolic compound degradation was witnessed when B-TiO_2_ and Mn-B-TiO_2_ NPs were suspended in TPOME solution and irradiated with visible light, suggesting the appropriate photocatalytic activity of the samples. The B-TiO_2_ removed 48.17% of phenolic compounds from TPOME with an apparent rate constant of 0.2865 min^−1^.

On the other hand, the photocatalytic removal efficiency of phenolic compounds by Mn-B-TiO_2_ NPs reached a maximum of 60.12% from 48.17% at 0.3 wt% Mn doping concentration with an apparent rate constant of 0.3992 min^−1^. The pseudo first order kinetics of phenol degradation are shown in Figure 7c. Initially, with Mn doping as the Mn wt% increased to 0.1 and 0.3%, the phenolic compounds’ removal efficiency from TPOME continued to grow and afterward decreased when the Mn doping level was raised to 0.5 wt%. This is consistent with the work by Deng et al. [50], who studied the effect of the Mn doping concentration on the photocatalytic removal of methylene blue under visible-light irradiation. Their results showed that the 0.2% Mn doped sample was the most active, showing 90% methylene blue removal in 210 min of visible-light irradiation. The disparity in the results compared to previous works is probably due to the complex nature of the TPOME matrix, which contains several organic molecules. Previous works have tested the Mn-doped TiO_2_ to decompose a single model compound in aqueous suspension rather than real environmental matrices.

In addition, an appropriate concentration of Mn induced intermediate bands as a steppingstone for the generation of charge carriers under the energy of various wavelengths. The associated transfers of high-charge carriers to the photocatalyst surface to perform photocatalytic reactions could be attributed to the curvy nature. This increased the life span of the electron–hole pair and resulted in higher photoactivity [50]. Furthermore, the higher photocatalytic performance of Mn-B-TiO_2_ for removing phenolic compounds from TPOME could also be attributed to the generation of more free charge carriers, which helped to induce surface chemical reactions under visible-light irradiation compared to undoped TiO_2_. However, when the doping concentration was above the optimum level, many crystal defects could be induced, which may have served as recombination centers for electron–hole pairs, thus reducing photoactivity [33]. Stucchi et al. [27] reported that the dopant type and their oxidation state are the key parameters that significantly influence the photocatalytic removal of organic contaminants under visible-light irradiation. Sclafani et al. [59] emphasized the need to determine the physicochemical properties since semiconducting properties and physicochemical properties determine the ultimate photocatalytic activity.

The previous investigation on the effect of Mn doping on photocatalytic activity rarely considered the textural properties of the Mn-doped TiO_2_ for the removal of organic pollutants. Various studies suggest a positive effect of different metals’ ions with various oxidation states (1+, 2+, 3+, and 4+) on TiO_2_-based photocatalysis [60]. Herein, the effect of the M^3+^ and Mn^4+^ ratio on the photocatalytic performance of Mn-doped TiO_2_ for the degradation of phenolic compounds from TPOME was investigated. Interestingly, it was found that the higher ratio of M^3+^/Mn^4+^ led to enhanced photocatalytic removal of phenolic compounds from TPOME. The photocatalytic removal efficiency of phenolic compounds reached ~60.12% at a ratio of 1.5 of M^3+^/Mn^4+^. This could be due to the highly charged Mn (M^3+^/Mn^4+^) ions inducing polarity on the TiO_2_ surface. This ratio was caused when the Mn doping level was 0.3 wt%.

Nevertheless, it was somewhat difficult to determine whether this positive effect is due to Mn^3+^/Mn^4+^ at a higher ratio or the specific surface area because the TiO_2_ samples doped with 0.3% Mn also showed a larger surface area compared to the sample doped with 0.5% Mn. To rule this out, we observed that the sample 0.1% Mn showed a higher surface area than 0.3%. However, its photocatalytic performance was lower compared to the 0.3% Mn-doped samples. Thus, the higher photocatalytic removal efficiency of phenolic compounds was probably due to the higher Mn^3+^/Mn^4+^ ratio and the cumulative effect of other factors such as lower bandgap, appropriate pore volume, and pore size.

In the current study, the photocatalytic removal efficiency of phenolic compounds reduced when the Mn concertation was further increased by 0.5%. The photocatalytic removal efficiency dropped to 24.20 at a 0.5% Mn doping level. The decrease in photocatalytic removal efficiency can be attributed to a decrease in surface area (~32%) upon increasing the Mn concertation to 0.5 wt%. High loading of Mn led to the formation of a complete layer of Mn^n+^ covering the surface of TiO_2,_ resulting in lower photon absorption or light penetration to reach the active sites, which impeded the photocatalytic activity.

### 3.3. Photocatalytic Degradation Mechanism

**Figure 8:**
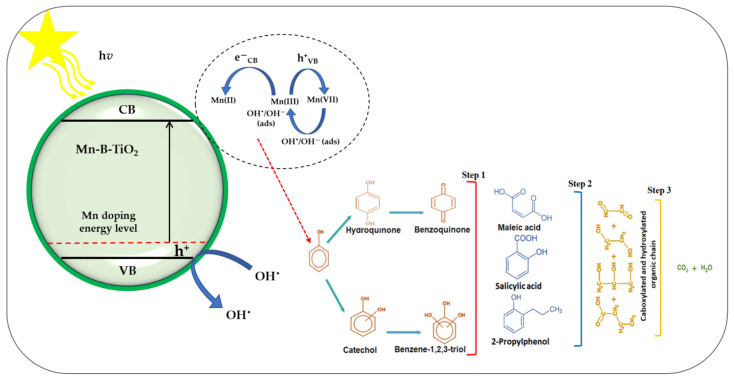
The photocatalytic degradation mechanism of phenols over Mn-B-TiO_2_ under visible light irradiation [64,65].

Figure 8 shows the proposed mechanism of photocatalytic degradation of phenols. As demonstrated by the characterization results, Mn modifies the photoelectric properties of B-TiO_2_ by producing an intermediate Mn 3d-O 2p energy level within its bandgap. Above the VB edge in Mn-B-TiO_2_, an intermediate energy level improves the separation of (e−/h+) pairs to facilitate the electron transfer and narrows the optical band gap to absorb more visible light by shifting the spectral photoresponse toward longer wavelengths. The half-filled electronic structure of Mn^x+^ enhances the photocatalytic activity because of its electronic configuration 3d^5^, which changes to d^4^ when accepting h+ and to d^6^ upon receiving electrons [61]. The energy level of the multiple Mn^3+^/Mn^4+^ is below the CB edge, while that of Mn^6+^/Mn^7+^ is above the VB [62]. Consequently, Mn(III) can trap both h+ and electrons to assist the charge transfer process in TiO_2_. Mn(IV) is formed via the oxidation of Mn(III) by h+ through a valence band process, and then free hydroxyl OH^−^ is quickly adsorbed and reacts with unstable Mn(III) to produce adsorbed radicals [63].

The hydroxyl radical then attacks phenol molecules. Moreover, the mechanism described here or in the literature is confirmed by C-13 tracking. The results of carbon-13 tracking, as listed in the References, show hydroquinone, resorcinol, and maleic acid as the major intermediates of degradation [64,65]. The degradation of phenol occurs via the substitution of aromatic dihydroxy followed by the aromatic ring cleavage and decarboxylation of the organic chain to mineralization. The predominant degradation pathway is the hydroxyl radical mechanism, as shown by the distribution of intermediates formed.

### 3.4. Electrical Energy Consumption

Table 3 shows the calculated EE required for the photocatalytic process to remove phenol from TPOME using various types of photocatalyst. The treatment cost was calculated based on the Islamic Republic of Malaysia’s energy cost per unit of 0.053 USD per kWh as of October 2021. With an apparent rate constant of 0.2865 min^−1^, the EE consumed after 180 min of the visible-light-driven photocatalytic process utilizing B-TiO_2_ was 104.71 kWh/m^3^, resulting in a 52.48% decrease in phenol concentration with a cost of 5.54963 USD per unit of TPOME treatment. The 0.3 wt% Mn-B-TiO_2_ used just 75.15 kWh/m^3^ of EE with an apparent rate constant of 0.3992 min^−1^, resulting in a 76.05% reduction in phenol concentration with a cost per unit volume of TPOME treatment of 3.98295 USD.

This study found that Mn-B-TiO_2_ consumed the least amount of EE and cost less than the other samples as well as the prices identified in pre-existing studies [4,28]. The prolonged treatment time (>300 min) in previous research may be to blame (180 min). In addition to reducing phenol content by 60%, the photocatalytic method based on Mn-B-TiO_2_ minimized the amount of EE needed to treat one unit volume of wastewater. The treatment cost was calculated based on the Islamic Republic of Malaysia’s energy cost per unit of 0.053 USD per kWh as of October 2021. The treatment cost per cubic meter of the phenolic wastewater by B-TiO_2_ and Mn-B-TiO_2_ NPs was 5.54963 and 3.98295 USD, respectively, which was the lowest of the photocatalysts examined in this study.

### 3.5. Recyclability and Stability Tests

**Figure 9:**
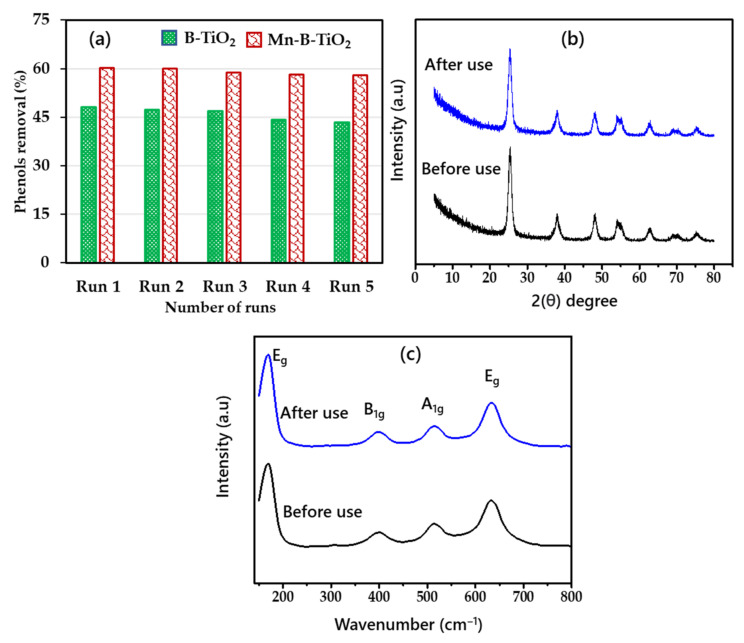
(**a**) Recyclability tests results of B-TiO_2_ and Mn-B-TiO_2_ NPs, (**b**) XRD patterns, (**c**) Raman spectra of Mn-B-TiO_2_ NPs.

The recyclability of the spent B-TiO_2_ and Mn-B-TiO_2_ NPs was evaluated for the degradation of phenols from actual wastewater, TPOME. The spent NPs were recovered from the treated TPOME solution after the photoreaction. Subsequently, the recovered B-TiO_2_ and Mn-B-TiO_2_ NPS were washed with deionized water and dried in an oven at 80 °C for 12 h before being used in the next run. Figure 9a shows the recyclability results. The effectiveness of the B-TiO_2_ was significantly reduced compared to Mn-B-TiO_2_. The phenols’ removal efficiency of the B-TiO_2_ reduced from 48.17 to 43.48% after the fifth run. On the other hand, the phenols’ removal efficiency by Mn-B-TiO_2_ lowered from 60.12 to 57.98% after the fifth cycle, suggesting the better recyclability of the sample. The results suggest that Mn-B-TiO_2_ has higher recyclability. The spent Mn-B-TiO_2_ NPs were characterized using XRD, and Raman spectroscopy was used after the reaction to determine the stability of the synthesized material.

The XRD patterns of Mn-B-TiO_2_ NPs before and after the reactions are shown in Figure 9b. No significant decrease in intensity and broadening of the anatase peak can be seen in the XRD patterns (101 plane), suggesting that the anatase crystalline structure of the material was unchanged after 180 min of visible light reaction. Figure 9c shows the Raman spectra of CS-TiO_2_ before and after the treatment. The spectra revealed four main Raman bands at 168.12 cm^−1^ (E_g_), 399.49 cm^−1^ (B_1g_), 513.10 cm^−1^ (A_1g_), and 632.43 cm^−1^ (E_g_), all of which were associated with anatase phase [66] and were comparable with the XRD results. The stronger vibration mode at 168.12 cm^−1^ (E_g_), corresponding to the Ti—O bending vibrations [67,68], showed no change, indicating that the Mn-B-TiO_2_ NPs were very stable under the studied conditions.

The better recyclability and higher stability of the Mn-B-TiO_2_ can be attributed to the higher Oxidation states Mn(IV)-Mn(VII) over Mn(II) because it was previously revealed that the greater positive charge added over the surface of TiO_2_ due to higher Mn ions offers a great deal of photocatalyst stability due to the generation of repulsive interactions among the nanoparticles [23]. Mn(IV)-Mn(VII) is not soluble in water and has higher oxidation power than Mn(II). This has facilitated the functioning of Mn-B-TiO_2_ in real wastewater matrices under light irradiation without leaching out of the impregnated Mn. Furthermore, it has been reported that impregnating higher oxidation states of Mn (Mn^4+^) not only provides stability but also enhances the adsorption and photodegradation ability of TiO_2_. On the other hand, the Mn^2+^ state is not suitable for applications in wastewater treatment since it is not stable and will leach out from the photocatalyst.

## 4. Conclusions

The photocatalytic efficacy of black TiO_2_ and black TiO_2_ modified with Mn was tested in this work to remediate treated palm oil mill effluent. The optical characteristics of Mn-modified black TiO_2_ nanoparticles were found to be good. The bandgap of black TiO_2_ was significantly reduced when doped with 0.5 wt% Mn, and the visible light absorption was drastically enhanced. The electron–hole recombination was successfully suppressed in Mn-modified black TiO_2_ nanoparticles. The surface morphology was ameliorated with Mn doping, whereas the textural properties moderately deteriorated. The 0.3 wt% Mn-modified black TiO_2_ nanoparticles were able to remove 60.12% of the phenols from treated palm oil mill effluent with an apparent rate constant of 0.3992 min^−1^, as compared to only 48.17% with a precise rate constant of 0.2865 min^−1^ for black TiO_2_ nanoparticles. The Mn modification of black TiO_2_ nanoparticles reduced the treatment cost by 32.31% from 5.54963 to 3.98295 USD per order of magnitude for TPOME.

## Figures and Tables

**Table 1:** Summary of the photocatalytic properties of the B-TiO_2_ and Mn-B-TiO_2_ NPs.

Properties	B-TiO_2_	Mn-B-TiO_2_ (0.1%)	Mn-B-TiO_2_ (0.3%)	Mn-B-TiO_2_ (0.5%)
FWHM	0.5872	0.5628	0.5117	0.4093
d-spacing (Å)	3.498	3.507	3.504	3.550
Crystallite size (nm)	49.31	47.63	47.89	48.41
Crystallinity (%)	60.82	46.00	47.49	45.79
Absorption edge (nm)	550	>600	>600	>600
Bandgap	2.96	2.49	2.30	2.21
Specific surface area (m^2^/g)	99.88	97.35	94.03	74.60
Pore volume (m^3^/g)	0.198	0.216	0.210	0.231
Pore size (nm)	3.06	5.01	5.03	5.08

**Table 2:** XPS fitting parameters for B-TiO_2_ and Mn-B-TiO_2_ NPs.

Fitting Parameters	Binding Energy (eV)	
	B-TiO_2_	Mn-B-TiO_2_ NPs
Ti2p_3/2_ (Ti^4+^)	459.04	460.04
Ti2p_1/2_ (Ti^3+^)	460.46	458.70
Ti2p_3/2_ (Ti^4+^)	465.05	465.58
Ti2p_1/2_ (Ti^3+^)	—	—
^1^ O_L_	529.94	530.31
^2^ O_V_	531.37	531.56
C–C	284.71	285.08
C–O	—	285.88
Coke-C	288.78	289.24
Mn^3+^	—	642.65
Mn^4+^	—	643.98
Mn^6+^	—	645.41
Mn^7+^	—	646.28

^1^ lattice oxygen, ^2^ oxygen vacancy.

**Table 3:** Apparent rate constants, and electrical energy consumption by different photocatalysts.

Photocatalyst	Phenol Removal (%)	K_app_ (min^−1^)	Electrical Energy (kWh/m^3^)	Cost USD/m^3^
B-TiO_2_	48.17	0.2865	104.71	5.54963
Mn-B-TiO_2_ (0.1%)	52.37	0.3221	93.14	4.93642
Mn-B-TiO_2_ (0.3%)	60.12	0.3992	75.15	3.98295
Mn-B-TiO_2_ (0.5%)	23.97	0.1190	252.11	13.36183

## Data Availability

Not applicable.
